# Characterization of the Proteolytic Activity of a Halophilic *Aspergillus reticulatus* Strain SK1-1 Isolated from a Solar Saltern

**DOI:** 10.3390/microorganisms10010029

**Published:** 2021-12-24

**Authors:** Dawoon Chung, Woon-Jong Yu, Ji-Yeon Lim, Nam-Seon Kang, Yong-Min Kwon, Grace Choi, Seung-Sub Bae, Kichul Cho, Dae-Sung Lee

**Affiliations:** 1 Department of Genetic Resources Research, National Marine Biodiversity Institute of Korea, Seocheon 33662, Korea; dwchung@mabik.re.kr (D.C.); woonjong_yu@mabik.re.kr (W.-J.Y.); lim87929@mabik.re.kr (J.-Y.L.); jichi9@mabik.re.kr (Y.-M.K.); gchoi@mabik.re.kr (G.C.); ssbae@mabik.re.kr (S.-S.B.); kichul.cho@mabik.re.kr (K.C.); 2 Department of Taxonomy and Systematics, National Marine Biodiversity Institute of Korea, Seocheon 33662, Korea; kang3610@mabik.re.kr

**Keywords:** halophilic fungi, saltern, protease, *Aspergillus reticulatus*

## Abstract

Salterns are hypersaline environments that are inhabited by diverse halophilic microorganisms, including fungi. In this study, we isolated a fungal strain SK1-1 from a saltern in the Republic of Korea, which was identified as *Asperillus reticulatus*. This is the first reported saline-environment-derived *A. reticulatus* that belongs to the *Aspergillus penicillioides* clade and encompasses xerophilic fungi. SK1-1 was halophilic, obligately requiring NaCl for growth, with a maximum radial growth of 6%–9% (*w*/*v*) NaCl. To facilitate the biotechnological application of halophilic fungi, we screened the SK1-1 strain for proteolytic activity. Proteases have widespread applications in food processing, detergents, textiles, and waste treatment, and halophilic proteases can enable protein degradation in high salt environments. We assessed the proteolytic activity of the extracellular crude enzyme of SK1-1 using azocasein as a substrate. The crude protease exhibited maximum activity at 40–50 °C, pH 9.5–10.5, and in the absence of NaCl. It was also able to retain up to 69% of its maximum activity until 7% NaCl. Protease inhibitor assays showed complete inhibition of the proteolytic activity of crude enzymes by Pefabloc^®^ SC. Our data suggest that the halophilic *A. reticulatus* strain SK1-1 produces an extracellular alkaline serine protease.

## 1. Introduction

Microorganisms in extreme environments have gained increasing scientific attention because they serve as valuable models of functional evolution and have potential biotechnological applications [1,2]. During physiochemical adaptation to harsh environments, they can obtain unique genetic and metabolic traits, as compared to microorganisms in general environments [3].

Solar salterns are extreme hypersaline environments that are inhabited by a diverse range of microorganisms, including archaea, bacteria, and fungi [4]. Several halophilic and halotolerant fungal species isolated from solar salterns have been previously reported. Fungi prevalently isolated from salterns include black yeasts (*Hortaea werneckii* and *Aureobasidium pullulans*), *Aspergillus*, *Penicillium*, and *Cladosporium* species [5,6,7]. Moreover, *Wallemia ichthyophaga* and *Aspergillus penicillioides* have been reported as obligate halophilic fungi isolated from salterns [8,9].

Enzymes from extremophilic microorganisms exhibit several useful properties, including thermostability, pH and salinity tolerance, and the ability to catalyze reactions in non-standard environments. To discover more effective and efficient microbial enzymes and to facilitate the application of halophilic microorganisms, our laboratory screened fungi from saline environments for enzymatic activities. Proteases hydrolyze the peptide bonds of proteins and these account for more than 65% of global enzyme sales [10]. Microbial proteases are important in food processing, leather, textile, and detergent industries, and in waste treatment applications. Among fungi, *Aspergillus*, *Penicillium*, *Rhizopus*, *Mucor*, and *Humicola* species are used for protease production [11].

In this study, we aimed to provide novel and fundamental information of halophilic fungal species and its protease activity. We isolated a fungal strain from a hypersaline saltern, designated as SK1-1, which required NaCl for its growth and exhibited proteolytic activity. We studied the halophilic growth of SK1-1 and identified this strain using morphological and phylogenetic analyses. In addition, protease activity of the extracellular crude enzyme of SK1-1 was characterized using azocasein as a substrate.

## 2. Materials and Methods

### 2.1. Sample Collection, Isolation, and Cultivation of Fungi

Seawater was collected from ponds in the Sekwang solar saltern in the Republic of Korea (34°36′39.54″ N, 126°17′22.52″ E). Seawater was filtered using a 0.45 µm membrane filter (Hyundai Micro Co., Seoul, Korea), and the filters were placed on Czapek-Dox agar (CDA, BD, Franklin Lakes, NJ, USA), malt extract agar (MEA, Oxoid, Hampshire, UK), and potato dextrose agar (PDA, BD) containing 5% or 10 % (*w*/*v*) NaCl (Oxoid), 0.01% (*w*/*v*) ampicillin (Sigma, St. Louis, MO, USA), and 0.01% (*w*/*v*) streptomycin (Sigma). Following incubation at 20 °C for 7–14 days, individual fungal colonies were picked from the plates and were repeatedly streaked on fresh media in order to obtain pure cultures. To identify halotolerant or halophilic fungi, fungal isolates were cultured on PDA and supplemented with 0%, 5%, and 10% NaCl at 20 °C for 7–21 days. Fungal isolates showing growth on the PDA containing 5% and 10% NaCl, but not on 0% NaCl, were selected for further study. Fungal spores and hyphal fractions were suspended in 20% glycerol (*v*/*v*) and stored at −80 °C for long-term storage.

### 2.2. Optimization of NaCl Concentrations for Radial Growth of SK1-1

To determine the optimum NaCl concentration of SK1-1 growth, three distinct media, including Czapek Dox broth (CDB, BD), malt extract broth (Oxoid), and potato dextrose broth (PDB, BD) were used. Each medium was prepared according to the manufacturer’s instructions, followed by the addition of 0%, 3%, 6%, 9%, 12%, 15%, or 20% NaCl. The pH was adjusted to 6 using NaOH (Sigma) or HCl (Sigma) in all the media, and 1.5% (*w*/*v*) Bacto agar (BD) was added for solidification. After autoclaving at 120 °C for 20 min, 15 mL of each medium was poured into a Petri dish and solidified. A conidial suspension in sterile water (5 µL, containing 10^5^ conidia) was spot inoculated on the media. Following incubation at 28 °C for 5 days, the diameter of each colony was measured. This experiment was performed in triplicate.

### 2.3. Identification of SK1-1

For micromorphological identification, SK1-1 was inoculated on agar plugs (approximately 10 mm × 10 mm) of PDA containing 6% NaCl, and coverslips were mounted onto the plugs. Following cultivation at 28 °C for 5 days, conidia and conidiophores were observed under a microscope (CTR6000, Leica, Wetzlar, Germany). Images were obtained using a Leica DMC2900 camera and LAS software (version 4.5, Leica).

For molecular identification, sequences of two genetic markers, i.e., ribosomal internal transcribed spacer (ITS) and beta-tubulin (BenA), were analyzed. To isolate fungal genomic DNA (gDNA), SK1-1 was cultured in PDB at 28 °C and 200 rpm for 3 days. Mycelia were collected on sterile Miracloth (Millipore, Billerica, MA, USA), frozen in liquid nitrogen, and ground to a fine powder using a mortar and pestle. The remaining procedures for gDNA isolation were performed as described previously [12].

The two individual DNA fragments (ITS and BenA) were amplified from gDNA using the polymerase chain reaction (PCR). The ITS region containing ITS-1, 5.8S rRNA, and ITS-2 was amplified using the primers ITS1 and ITS4 [13]. The BenA fragment was amplified using primers Bt2a and Bt2b [14]. PCR conditions were as follows: 95 °C for 3 min, followed by 34 cycles of 30 s at 95 °C, 30 s at 55 °C, and 1 min at 72 °C, and a final extension at 72 °C for 15 min. PCR products were purified using a gel extraction kit (Qiagen, Hilden, Germany) and sequenced as previously described [12].

### 2.4. Phylogenetic Analysis

The obtained sequences of ITS and BenA were used as a query for a BLAST search to identify sequences closely related to SK1-1 in the GenBank database. The sequences were aligned and adjusted using MEGA 6 software [15]. Phylogenetic trees were generated using the neighbor-joining method, and confidence in the branches was assessed by bootstrap analysis with 1000 replicates in MEGA 6.

### 2.5. Skim Milk Agar Assay for Proteolytic Activity

The proteolytic activity of SK1-1 was qualitatively assessed using skim milk (BD) agar. Skim milk medium was prepared by combining 1% (*w*/*v*) skim milk, 0.01% (*v*/*v*) Triton X-100 (Sigma), and 1.5% (*w*/*v*) Bacto agar with CDB. SK1-1 conidia were inoculated on the skim milk medium and cultured at 28 °C for 7 days. Proteinase K (Qiagen) and sterile H_2_O were used as positive and negative controls, respectively. Proteolytic activity was determined based on the presence of clear zones around the colonies.

### 2.6. Preparation of the Extracellular Crude Enzyme

For the quantitative assessment of proteolytic activity, extracellular crude enzymes were collected from the liquid cultures of SK1-1. First, SK1-1 was cultured on PDA containing 6% (*w*/*v*) NaCl at 28 °C for 7 days. Then, ten agar plugs were obtained from the PDA plates using a sterile cork borer and inoculated in 50 mL skim milk broth (0.5% [*w*/*v*] skim milk, 0.01% [*v*/*v*] Triton X-100, 6% [*w*/*v*] NaCl in CDB) and cultured at 28 °C and 100 rpm for 14 days. The SK1-1 supernatants were collected by filtering through Miracloth and were sterilized using syringe filtration (0.22 μm pore size). These cell-free supernatants were used as the extracellular crude enzyme, and the amount of total protein in the enzyme solution was measured using the Protein Assay Dye Reagent Concentrate (Bio-Rad, Hercules, CA, USA). The crude enzyme was stored at −20 °C until used.

### 2.7. Proteolytic Activity Assays Using Azocasein as a Substrate

Azocasein (Sigma) was used as a substrate to measure proteolytic activity, as previously described [16,17] with some modifications. An aliquot of the crude enzyme solution (50 µL, containing 10 μg protein) was mixed with the azocasein solution (3 mg/mL in 20 mM phosphate buffer pH 6.0), followed by incubation at 37 °C for 2 h. The enzymatic reaction was stopped by adding 5% (*w*/*v*) trichloroacetic acid (TCA, Sigma) solution to the mixture. Following centrifugation at 97,000× *g* for 10 min, 100 μL of the supernatant was transferred to a 96-well plate. A 0.5 N NaOH (Sigma) solution was added to the well at a ratio of 1:1 (*v*/*v*), and the optical density was measured at 440 nm (OD_440_) using a microplate reader (HIDEX, Turku, Finland) against a reaction blank containing the reaction mixture with TCA-inactivated enzyme. Proteolytic activity was calculated as the amount of digested azocasein by the extracellular crude enzyme per hour.

A calibration curve was obtained by plotting the OD_440_ against the azocasein concentration [16]. To determine the incubation time (t_max_) in order to achieve the maximum optical density at 37 °C, azocasein solutions at concentrations of 0.5, 1.5, 3.0, and 5.0 mg/mL were mixed with the crude enzyme and the OD_440_ was measured every 2 h for 24 h. Then, the calibration curve was generated by plotting the azocasein concentrations at t = 0 (initial time point) and the optical densities at t_max_, as previously described [16].

### 2.8. Influence of Temperature, pH, and NaCl Concentration on Proteolytic Activity

To determine the optimum temperature for proteolytic activity, the enzyme-substrate reaction mixture was incubated at 10, 20, 30, 40, and 50 °C for 1 h. To determine the optimum pH, the azocasein solutions at distinct pH values were prepared using distinct buffers: acetic acid-sodium acetate (pH 5.5), phosphate (pH 6.5 and pH 8.0), and glycine-NaOH (pH 9.5 and pH 10.5). To investigate the optimum NaCl concentration, 0%, 1.5%, 3%, 5%, 7%, and 10% (*w*/*v*) NaCl was added to the azocasein and dissolved in 20 mM phosphate buffer (pH 6.0), and the pH was adjusted to pH 6.0 using NaOH or HCl for assaying proteolytic activity.

### 2.9. Influence of Metal Ions on Proteolytic Activity

The effects of three metal ions (Ca^2+^, Mg^2+^, and Zn^2+^) on the activity of the SK1-1 crude protease were examined using CaCl_2_, MgSO_4_, and ZnSO_4_, respectively (Sigma). Each compound was mixed with the crude enzyme at final concentrations of 2 and 4 mm and incubated for 15 min at room temperature (23 °C). Then, the azocasein solution was added to the mixture, followed by incubation at 37 °C for 2 h. Proteolytic activity was measured, as described above. The activity, in the absence of metal ions, was considered to be the control (100% activity).

### 2.10. Determination of Thermal Stability of Proteolytic Activity

Thermostability was determined by incubating the crude enzyme at 10, 20, 30, 40, and 50 °C for 1 h prior to the enzyme reaction. After cooling on ice, the crude enzyme was reacted with azocasein (3 mg/mL) at 37 °C for 2 h. The proteolytic activity of the crude enzyme, without preincubation, was considered to be the control (100%), and the residual activities of each reaction mixture were measured.

### 2.11. Protease Inhibitor Assay

The effect of protease inhibitors on the proteolytic activity of the SK1-1 crude enzyme was examined using three individual protease inhibitors from the Protease Inhibitors Set (Sigma). The crude enzymes were treated with leupeptin (at working concentrations of 1.5 and 3 μg/mL), pepstatin (PS; 7.5 and 15 μg/mL), and Pefabloc^®^ SC (PSC; 0.5 and 1 mg/mL). The solution of each protease inhibitor was prepared in appropriate solvents according to the manufacturer’s instructions. The crude enzyme was mixed with each protease inhibitor and incubated at room temperature (23 °C) for 30 min. Then, the mixture was reacted with azocasein at 37 °C for 2 h. The proteolytic activity of the crude enzyme without protease inhibitor treatment was considered to be the control (100%).

### 2.12. Statistical Analysis

Data were analyzed using the Prism software (version 5.0, GraphPad Software, San Diego, CA, USA). All experiments were performed in biological triplicate. One-way ANOVA, followed by a Tukey’s multiple comparison test or a *t*-test, was performed, and the significance level was set at 5%.

## 3. Results

### 3.1. Halophilic Growth of SK1-1

The temperature and salinity of the pond water of saltern were 31.5 °C and 46.3‰, respectively. When the fungi were cultured on PDA supplemented with 0%, 5%, and 10% NaCl to isolate pure colonies, we observed that one strain, designated SK1-1, did not grow on PDA without NaCl supplementation. However, it grew on PDA supplemented with 5% and 10% NaCl, suggesting that this strain requires NaCl for growth, and is halophilic.

**Figure 1:**
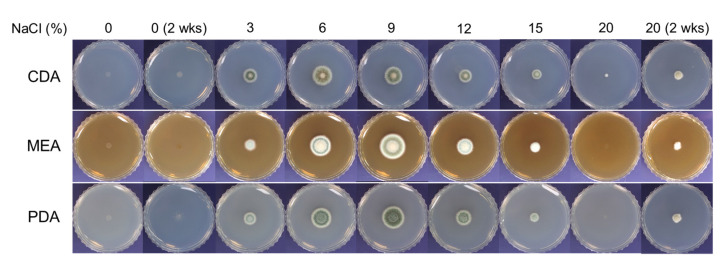
Halophilic growth of SK1-1. Conidia of SK1-1 (10^5^ conidia in sterile water) were inoculated on Czapek Dox agar (CDA), malt extract agar (MEA), and potato dextrose agar (PDA) containing 0%, 3%, 6%, 9%, 12%, 15%, and 20% (*w*/*v*) NaCl. Following incubation at 28 °C for 5 days or 2 weeks (2 wks), colony growth and diameter were compared to optimize the NaCl concentration for maximum radial growth of SK1-1.

The growth of SK1-1 on media containing 0% or 20% NaCl was extremely limited when cultured at 28 °C for 5 days (Figure 1). During prolonged incubation for 2 weeks, slightly improved growth of SK1-1 was observed on the media supplemented with 20% NaCl, whereas the growth was still restricted on media without NaCl.

The colony size (diameter) of SK1-1 gradually increased during growth on MEA and PDA containing 3% to 9% NaCl. It peaked at 9% (the optimum concentration) and decreased thereafter until reaching 20% NaCl. On CDA, the colony size increased from 3% to 6% NaCl. It peaked at 6% (the optimum concentration) and decreased thereafter (Figure 1). Under the optimum NaCl concentrations for growth, SK1-1 colonies on CDA, MEA, and PDA exhibited diameters of 1.67 ± 0.07 cm, 2.23 ± 0.03 cm, and 1.80 ± 0.06 cm, respectively.

### 3.2. Identification of SK1-1

**Figure 2:**
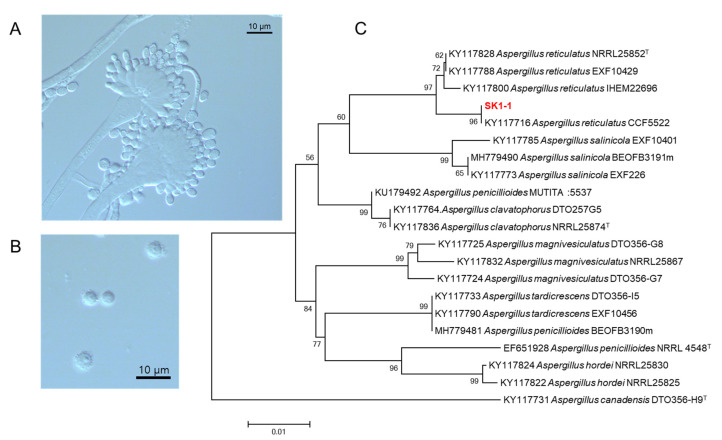
Morphological characterization and phylogenetic analysis of SK1-1. (**A**,**B**) Microscopic images of SK1-1 (**A**) conidiophores and (**B**) conidia. Scale bar = 10 μm. (**C**) Phylogenetic tree of SK1-1 obtained by a neighbor-joining analysis of β-tubulin sequences. Numbers at nodes indicate the percent bootstrap values from 1000 replicates (values < 50% are not shown). The scale bar indicates the number of nucleotide substitutions per site. “T” next to the strain name indicates the strain type of the species.

SK1-1 produced globose, subglobose, and tuberculate conidia, which formed on a single layer of sterigmata (uniseriate) (Figure 2A,B). The SK1-1 conidia were 3.8 ± 0.1 µm wide and 4.3 ± 0.1 µm long (N = 10).

For molecular identification, the ITS and β-tubulin sequences (GenBank accession number OL691116 and OL770139, respectively) were analyzed. Based on the NCBI BLASTn search results, the SK1-1 ITS sequence (418 nucleotides) was 100% identical to those of *Aspergillus reticulatus* CCF5518 and *A. penicillioides* KH00279 and HNC15-78. Moreover, it was 99.8% and 98.1% identical to the ITS sequences of *A. reticulatus* NRRL 25852 (type strain) and *Aspergillus salinicola* EXF 226, respectively. In a neighbor-joining phylogenetic tree constructed using the ITS sequence (Supplementary Figure S1), SK1-1 was grouped with both *A. reticulatus* and *A. penicillioides*. Therefore, identification of SK1-1 at the species level was not possible on the basis of the ITS sequence.

Based on the BLASTn search results, the β-tubulin of SK1-1 (362 nucleotides) shared 100% and 98.6% sequence identity with those of *A. reticulatus* CCF5522 and NRRL25852 (type strain), respectively. Moreover, it shared 94.3%, 94.0%, and 93.8% identity with the β-tubulin sequences of *A. penicillioides* MUT<ITA>:5537, *A. clavatophorus* DTO257G5, and *A. salinicola* EXF226, respectively. A neighbor-joining phylogenetic analysis using the β-tubulin sequence placed SK1-1 in the same group as *A. reticulatus* CCF5522, supported by a 96% bootstrap value (Figure 2C). Together, the results from the morphological and phylogenetic analyses support the hypothesis that SK1-1 is *A. reticulatus*.

### 3.3. Skim Milk Agar Assay of SK1-1

**Figure 3:**
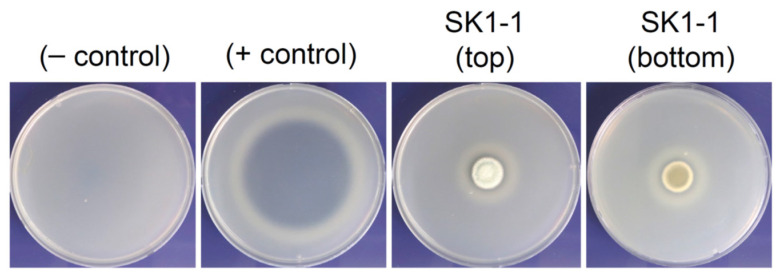
Skim milk agar assay for proteolytic activity of SK1-1. Skim milk (1%, *w*/*v*) was used as a substrate for protease. Sterile H_2_O and proteinase K were included as negative (−) and positive (+) controls, respectively. SK1-1 conidia were inoculated on skim milk agar, followed by incubation at 28 °C for 7 days. Proteolytic activity was determined by the presence of clear zones surrounding the colonies.

The turbidity of skim milk did not change with H_2_O (negative control), whereas a transparent zone appeared after inoculation with proteinase K (positive control). Like the positive control plate, incubation of SK1-1 on skim milk agar resulted in the formation of a transparent zone that surrounded the colony, thus suggesting that skim milk in that area was hydrolyzed by the protease of SK1-1 (Figure 3).

### 3.4. Construction of Calibration Curve for Azocasein Assays

Faster hydrolysis was observed at lower concentrations of azocasein than at higher concentrations (Supplementary Figure S2). For example, 0.5 and 1.5 mg/mL azocasein appeared to be completely digested within 10 h. In contrast, hydrolysis of 3.0 and 5.0 mg/mL azocasein reached the maximum optical density at time points between 18 and 24 h. As a result, we determined t_max_ as 24 h in order to generate the calibration curve for the quantification of proteolytic activity using azocasein.

### 3.5. Influence of Temperature, pH, and NaCl Concentration on Proteolytic Activity of SK1-1

**Figure 4:**
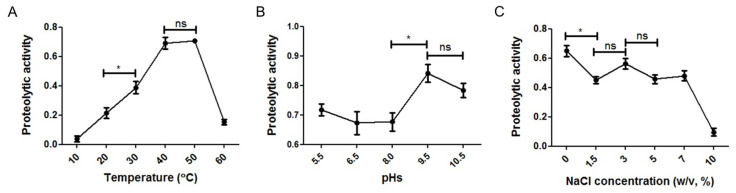
Influence of temperature, pH, and NaCl concentration on the proteolytic activity of SK1-1. Optimum temperature, pH, and NaCl concentration for proteolytic activity were determined with azocasein as substrate. All the experiments were performed in triplicate. ‘*’ and ‘ns’ indicate ‘significantly different (*p* < 0.05)’ and ‘not significantly different (*p* > 0.05)’, respectively, when two data sets were compared. Examination of optimum conditions of (**A**) temperature, (**B**) pH, and (**C**) NaCl concentration.

Overall, all these factors significantly affected the proteolytic activity of SK1-1 under the tested conditions (Figure 4). The maximum proteolytic activity of the SK1-1 crude enzymes was observed at 40 °C and 50 °C (Figure 4A). The activity increased with the rise in temperature from 10 °C to 40 °C, reaching a maximum at 40 °C and 50 °C, and then declined sharply between 50 °C and 60 °C. The pH optimum for proteolytic activity was observed under alkaline conditions (pH 9.5 and 10.5). The activity did not vary significantly from pH 5.5 to 8.0 (*p* > 0.05) (Figure 4B). The proteolytic activity on azocasein was at the maximum when no NaCl was added to the reaction mixture and remained relatively high when 1.5%–7% NaCl was added. However, a drastic decrease was observed with 10% NaCl addition (Figure 4C).

### 3.6. Effects of Metal Ions on Proteolytic Activity of SK1-1

In this study, we examined the effects of Ca^2+^, Mg^2+^, and Zn^2+^ on the proteolytic activity of SK1-1 using 2 mM and 4 mM CaCl_2_, MgSO_4_, and ZnSO_4_. Overall, the metal ions inhibited proteolytic activity, depending on their concentration. For example, the proteolytic activity was reduced by 4 mM Ca^2+^, Mg^2+^, and Zn^2+^ to approximately 72%, 88%, and 41%, respectively, as compared to the untreated control (Table 1).

### 3.7. Thermal Stability of the Crude Enzymes of SK1-1

**Figure 5:**
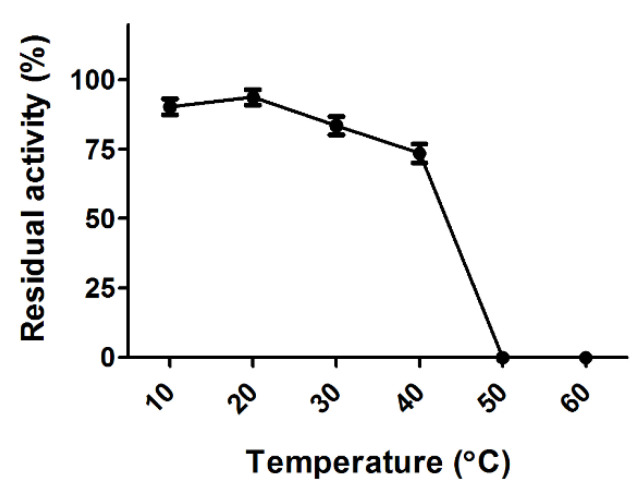
Thermal stability of the proteolytic activity of SK1-1. The crude protease was pre-incubated at temperatures ranging from 10 °C to 60 °C for 1 h prior to the enzymatic reaction. Residual proteolytic activity was assessed in comparison to that of the control (no pre-incubation).

The crude enzyme retained more than 90% of its initial proteolytic activity when pre-incubated at 10 °C and 20 °C for 1 h (Figure 5). The residual proteolytic activity reduced to 83.5% and 73.5% after incubation at 30 °C and 40 °C, respectively. In contrast, proteolytic activity was completely lost during preincubation at 50 °C and 60 °C for 1 h, suggesting that enzyme denaturation rapidly occurred between 40 °C and 50 °C.

### 3.8. Protease Inhibitor Assay

**Figure 6:**
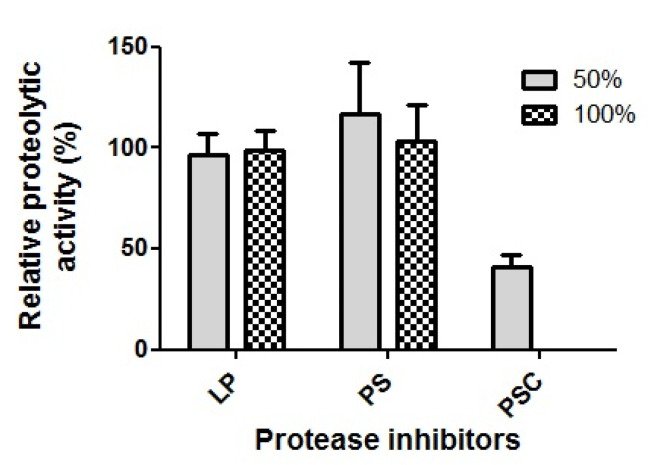
Effect of protease inhibitors on the proteolytic activity of SK1-1. The crude protease was treated with three individual protease inhibitors (LP, leupeptin; PS, pepstatin; PSC, Pefabloc^®^ SC) prior to the enzyme-substrate reaction. The terms ‘50%’ and ‘100%’ indicate the full and half amount of the manufacturer’s recommended dose, respectively. This experiment was performed in triplicate, and the results are expressed as mean values with standard errors.

LS (leupeptin, serine/cysteine protease inhibitor) and PS (pepstatin, aspartic protease inhibitor) did not affect the proteolytic activity of SK1-1 crude enzymes. In contrast, PSC (Pefabloc^®^ SC, serine protease inhibitor) significantly inhibited the proteolytic activity in a concentration-dependent manner. For example, the proteolytic activity reduced to approximately 41%, relative to the non-treated control in the presence of 0.5 mg/mL PSC, and the activity was completely inhibited by 1.0 mg/mL PSC (Figure 6).

## 4. Discussion

Depending on the NaCl concentration required for optimum growth, halophilic microorganisms can be categorized into three groups: slight (3% NaCl), moderate (3%–15% NaCl), and extreme (25% and no growth below 12% NaCl) halophiles [18,19]. Based on this definition and our results, SK1-1 appears to be a moderately halophilic fungus as well as an obligate halophile.

*A. reticulatus* was first reported in 2017, along with other xerophilic *Aspergillus* species [20]. It belongs to the *A. penicillioides* clade, which encompasses nine *Aspergillus* species. Based on phylogenetic distances, it is most closely related to *A. salinicola*. While *A. penicillioides* has been reported as an obligate halophile isolated from hypersaline environments [9], little is known about both *A. reticulatus* and *A. salinicola*. *A. reticulatus* was first isolated from diverse sources, such as lung biopsy, indoor and outdoor air, and carpet dust [20], and our study is the first report to describe saltern-derived (and ‘marine-derived’ in a broad sense) *A. reticulatus*.

There are several studies into halophilic fungi, which produce industrially important enzymes. The activities of amylase, cellulase, lipase, protease, and xylanase of halophilic fungal species isolated from hypersaline environments, including *A. flavus*, *A. gracilis*, *A. penicillioides*, *A. restrictus*, *Sterigmatomyces halophilus*, and *Gymnoascus halophilus* have been investigated [21,22]. Among the halophilic *Aspergillus* species, *A. gracilis* [23], *A. caesiellus* [24], *A. flavus*, and *A. restrictus* [21,25] exhibited amylolytic, lignocellulolytic, and proteolytic activities, respectively. However, the protease characteristics of *A. reticulatus* remains largely unknown.

The optimum temperatures of proteases originating from several *Aspergillus* species, including *A. foetidus*, *A. fumigatus*, *A. niger*, *A. oryzae*, and *A. terreus*, are in the range of 50–60 °C, which is similar to or slightly higher than that of SK1-1 (40–50 °C). In contrast, the pH optimum varies widely among these *Aspergillus* species. For example, the optimum pH for proteolytic activity was pH 2.5 in *A. niger* [26], pH 5.0–5.5 in *A. foetidus* and *A. oryzae* [27,28], and pH 9.0–9.5 in *A. fumigatus* and *A. terreus* [29,30].

According to the optimum pH for their activity, proteases can be divided into acidic (pH 3–4), neutral (pH 5–8), and alkaline (pH 8–12) proteases. The optimum pH of SK1-1 (pH 9.5–10.5) suggests that SK1-1 produces alkaline proteases that are similar to *A. fumigatus* and *A. terreus* [29,30]. Alkaline proteases are actively used in industrial processes that require alkaline conditions, such as detergents and leather processing [31]. Therefore, our data suggest that SK1-1 proteases also have the potential for similar applications.

The effects of the NaCl concentration on the proteolytic activity of halophilic fungi remain to be elucidated. In contrast, the protease activity of halophilic bacteria has been studied. The maximum proteolytic activities of moderately halophilic bacteria are observed at 6%, 1.2%–6%, and 18% NaCl in *Bacillus* sp., *Halobacillus karajensis*, and *Pseudomonas* sp., respectively [32,33,34]. Therefore, it might not be unusual for proteases and other enzymes from halophiles to possess high enzyme activities under salt-rich conditions. High salinity interrupts normal protein function by altering solubility, binding, stability, and the conformation of proteins [35]. Compared to general enzymes, the salt-adapted enzymes from halophilic microorganisms have the ability to maintain protein structure and activity under saline environments [36]. It is noteworthy that the protease activity of SK1-1 was examined using the crude enzyme, not a purified one in our study. Therefore, there is a possibility that the salt content in the crude enzyme could affect the determination of the optimum NaCl concentration for the protease activity of SK1-1.

Metal ions affect enzymatic activity by modulating the metal ion–protein interaction [37,38]. Effects of metal ions on protease activity vary in microbial proteases. For example, the activity of protease produced by *Bacillus* spp. increases by Mg^2+^ [39,40], whereas Mg^2+^ inhibits protease activity in an archaeon *Halogeometricum borinquense* [41]. While zinc ions have an inhibitory effect on the proteases produced by *Bacillus* sp. and *H. borinquense*, they increase protease activity in *Aspergillus niger* [39,40,42]. Generally, calcium ions have been reported to enhance protease activity [39,40,41,42]. However, our results showed that Ca^2+^ slightly inhibited the protease activity of SK1-1. One of the possible reasons for the difference is that the effect of calcium ions on the protease activity might depend on the concentration of Ca^2+^. Gupta et al. found that 1 mM Ca^2+^ increases, but 5 mM Ca^2+^ decreases the activity of the alkaline protease of *Bacillus* species [43]. Moreover, in *H. borinquense,* lower concentrations of Ca^2+^ (less than 25 mM) do not affect protease activity, although Ca^2+^ stimulates protease activity at higher concentrations [41].

Finally, proteases can also be categorized into several groups, such as aspartic, serine, and metalloproteases, depending on the nature of their active site [44]. The inhibited proteolytic activity of the SK1-1 crude enzyme by specific protease inhibitors suggests that SK1-1 produces a serine protease.

## 5. Conclusions

The halophilic features and proteolytic activity of SK1-1 characterized in this study provide novel information about saltern-derived *A. reticulatus*. Moreover, our experimental data suggest that the SK1-1 produces an extracellular alkaline serine protease with activity that remains relatively high over a broad range of NaCl concentrations. Given that microbial alkaline serine proteases account for approximately 89% of all commercial proteases [44], SK1-1 protease may have widespread applicability in various industries. To enable its biotechnological application, future work will require enzyme purification, culture optimization, and molecular studies.

## Figures and Tables

**Table 1:** Effect of metal ions on the proteolytic activity of SK1-1.

	Metal Ion Concentration	
Compounds	2 mM	4 mM
CaCl_2_	85.5 ± 6.9%	71.8 ± 5.6%
MgSO_4_	91.1 ± 4.6%	88.1 ± 5.0%
ZnSO_4_	59.7 ± 4.3%	40.8 ± 7.2%

The values indicate proteolytic activity (%) after treatment with individual metal ions relative to the untreated control. The activity of the untreated control was taken as 100%.

## Data Availability

The data presented in this study are available on reasonable request from the corresponding author.

## Supplementary Materials

The following are available online at https://www.mdpi.com/article/10.3390/microorganisms10010029/s1; Figure S1: Neighbor-joining phylogenetic analysis using ITS sequences. Numbers at nodes indicate percent bootstrap values from 1000 replications (values < 50% are not shown). Scale bar indicates the number of nucleotide substitutions per site. SK1-1 was grouped with *A. reticulatus* and *A. penicillioides*. “T” next to the strain name indicates the type strain of the species; Figure S2: Calibration curve for proteolytic assays using azocasein. The incubation time (t_max_) required to achieve the maximum optical density (OD_440_) in the proteolytic assay was determined at different azocasein concentrations (0.5, 1.5, 3.0, and 5.0 mg/mL), by measuring OD_440_ at 2-h intervals for 24 h.
