# Supplementary material for: Characterization of the Proteolytic Activity of a Halophilic Aspergillus reticulatus Strain SK1-1 Isolated from a Solar Saltern
Source: Microorganisms. 2021 Dec 24;10(1):29. doi: 10.3390/microorganisms10010029 (PMC8781784; doi:10.3390/microorganisms10010029)
Supplement: Supplementary file 1 [file microorganisms-10-00029-s001.zip › microorganisms-1484771-supplementary.pdf]

Supplementary Figure S1

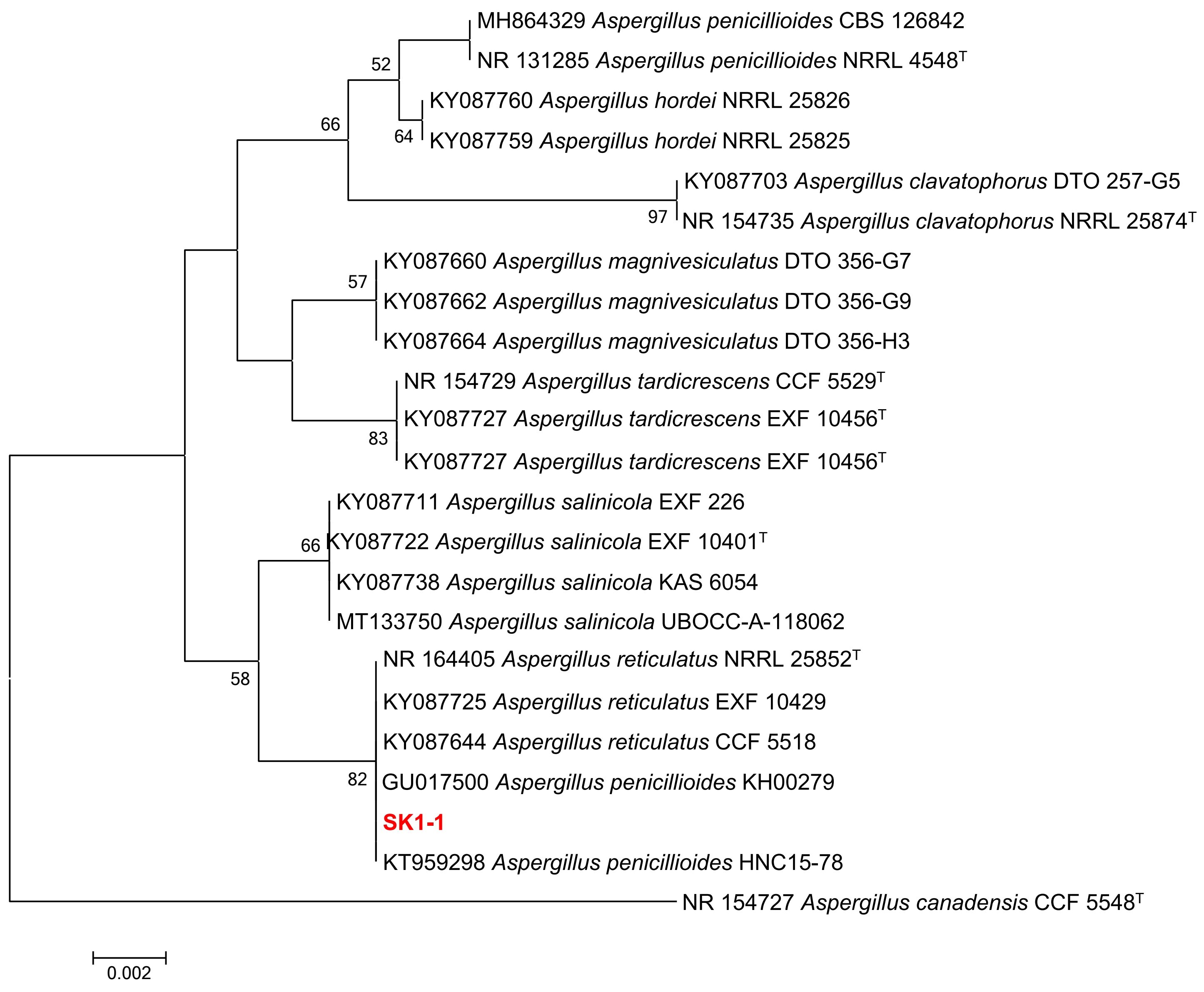

Supplementary Figure S1. A neighbor-joining phylogenetic analysis using ITS sequences
Numbers at nodes indicate percent bootstrap values from 1,000 replications (values <50% are not shown). The scale bar indicates the number of nucleotide substitutions per site. SK1-1 was grouped with *A. reticulatus* and *A. penicillioides*. "T" next to the strain name indicates a type strain of the species.

Supplementary Figure S2

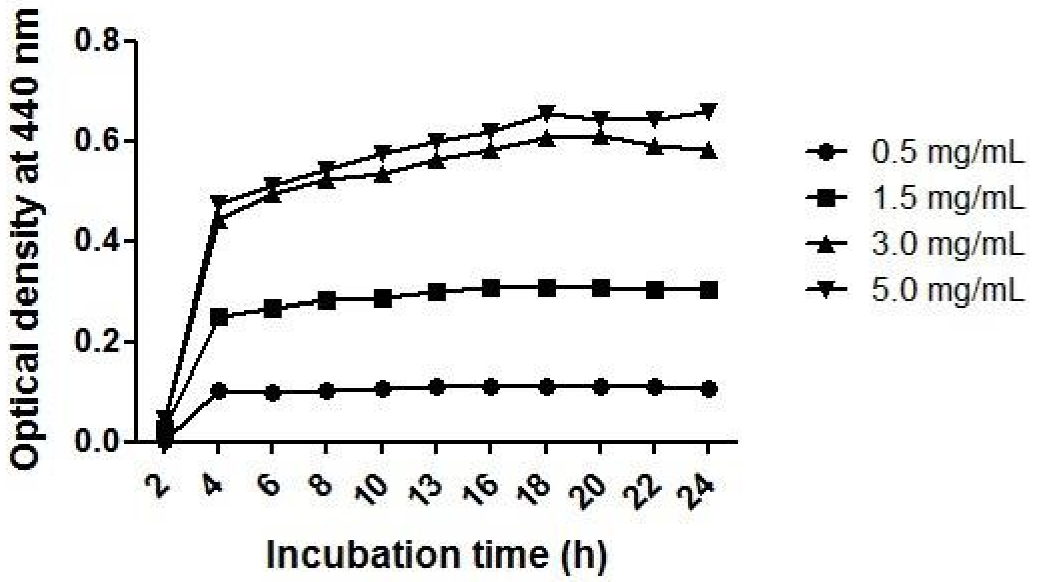

Supplementary Figure S2. Calibration curve for proteolytic assays using azocasein
The incubation time (t_{max}) to achieve the maximum optical density was determined by examining optical densities of a substrate-enzyme mixture at different azocasein concentrations (0.5, 1.5, 3.0, and 5.0 mg/mL). The optical densities were measured at 2-h intervals during 24 h.
